# Uncoupling traits, colonization, and benefits: genotype-dependent growth promotion by endophytic bacteria in Poaceae, including perennial Kernza

**DOI:** 10.1007/s11274-026-05405-y

**Published:** 2026-11-14

**Authors:** Jagoda Szydło, Bliss Ursula Furtado, Edyta Deja-Sikora, Sonia Szymańska, Katarzyna Hrynkiewicz

**Affiliations:** 1 Department of Microbiology, Faculty of Biological and Veterinary Sciences, Nicolaus Copernicus University, Lwowska 1, 87-100 Toruń, Poland; 2 Priority Research Team OBSIDIAN, Institute of Advanced Studies, Nicolaus Copernicus University in Toruń, Wileńska 4, 87-100 Toruń, Poland

**Keywords:** Bioinoculants, Endophytic bacteria, Host genotype effect, Poaceae, *Thinopyrum intermedium*, qPCR-based colonization assessment

## Abstract

*Thinopyrum intermedium* (Kernza) is a perennial Poaceae crop with potential benefits for soil health and contributes to climate change mitigation. In this study, we investigated whether bacterial functional traits and abundance predict growth responses across different Poaceae species. From 21 investigated bacterial strains representing endophytic and rhizosphere strains, five endophytes (*Labedella endophytica* FR-P-L-64, *Pseudomonas lini* FR-P-R2-32, *Pantoea* sp. GT2-2-ED1, *Pseudomonas stutzeri* ISE-12, *Streptomyces phaeochromogenes* ISE-4) were selected based on their plant-growth promoting (PGP) traits related to phytohormone production, nutrient assimilation, and stress alleviation. Phenotypic profiling revealed substantial metabolic diversity among the selected strains: *P. lini* FR-P-R2-32 showed the broadest spectrum of substrate utilization, while *S. phaeochromogenes* ISE-4 utilized only 10 of the 94 substrates. The strains were evaluated for their ability to promote plant growth in different Poaceae hosts, including two wheat cultivars, two perennial ryegrass varieties, and the perennial grain crop Kernza. Plant responses to inoculation were genotype-dependent: only *S. phaeochromogenes* ISE-4 increased shoot and root dry weight in Kernza and wheat cv. Euforia, whereas the remaining strains produced neutral or negative effects. Strain-specific quantitative PCR targeting the 16 S rRNA gene revealed distinct colonization patterns in shoots and roots including systemic, organ-specific, and limited colonization. Regression models indicated that post-inoculation endophyte abundance was positively or negatively associated with plant biomass, depending on the plant genotype. These findings highlight​ the importance of host genotype in determining the effectiveness of bacterial bioinoculants. This study provides the first functional characterization of bacterial endophytes isolated from *T*. *intermedium.*

**Supplementary Information:**

The online version contains supplementary material available at https://doi.org/10.1007/s11274-026-05405-y.

## Introduction

The need to mitigate the effects of climate change on agriculture has driven increasing interest in sustainable alternatives to conventional annual cropping systems (Crews et al. 2018; Kim et al. 2022). In this context, the perennial grain crop intermediate wheatgrass *Thinopyrum intermedium* (marketed as Kernza^®^) (DeHaan and Ismail 2017) has gained considerable attention due to its agronomic benefits and ecosystem services (Ginot et al. 2024). Its deep and dense root system confers high drought tolerance and efficient nutrient acquisition (Dietzel et al. 2015; Clément et al. 2022). Furthermore, cultivation of *T*. *intermedium* contributes to long-term soil health by reducing nitrogen fertilizer inputs by up to 50% (Jungers et al. 2017) and decreasing soil organic carbon losses by more than 38%, compared with annual crops (Tang et al. 2024). In addition, agricultural benefits of *T. intermedium* are associated with increased carbon input and root exudation, which stimulate soil microbial biomass, diversity, and activity (Bertola et al. 2023). *T. intermedium* belongs to the Poaceae family (commonly known as grasses), which comprises economically important cereal, pasture, and forage crops (Soreng et al. 2022).

Poaceae crops exhibit substantial genetic diversity at both the species and cultivar levels (Sow et al. 2025; MajidiMehr et al. 2025). In crops such as *Triticum aestivum* L. and *Lolium perenne* L., this diversity has been further expanded through breeding programs aimed at improving yield and tolerance to abiotic and biotic stresses (Bai et al. 2025; Saha et al. 2025). The application of bioinoculants to genetically diverse crops may be challenging because plant genotype strongly influences interactions with beneficial microorganisms (Wintermans et al. 2016; Foster et al. 2025). Host–microbe compatibility is largely determined by root exudate composition and plant immune responses (Poppeliers et al. 2023). Distinct plant genotypes produce characteristic root exudate profiles that shape the recruitment of specific microbial communities (Siebers et al. 2018; Fourneau et al. 2024). For example, previous studies observed that plant growth-promoting (PGP) bacteria exhibit differential chemotactic responses to root exudates released from rapeseed, pea, and ryegrass (Fourneau et al. 2024). Successful establishment within the host plant requires bioinoculants to modulate plant defense responses activated by the immune system (Deng et al. 2019; Yu et al. 2019). For instance, *Pseudomonas* strains can suppress root immunity in *Arabidopsis thaliana* by acidifying the pH levels in the rhizosphere (Yu et al. 2019).

Because the bioinoculant performance is strongly influenced by host genotype, there is a need to identify new microbial inoculants capable of promoting growth across a broad range of host plants. In this context, plant growth-promoting bacteria (PGPB) represent one of the most extensively studied groups of beneficial microorganisms (Kumar et al. 2022; Sharma et al. 2025). Among them, bacterial endophytes have attracted particular attention because they colonize internal plant tissues without causing disease symptoms, thereby potentially providing more direct and persistent benefits than rhizosphere-associated bacteria (Mushtaq et al. 2023; Ercole et al. 2025). PGPB enhance plant performance and stress resilience through multiple mechanisms, including indole-3-acetic acid (IAA) production, phosphate solubilization, siderophore synthesis, starch hydrolysis, and polyamine synthesis. These five key PGP-associated traits were investigated in our study to select potential bacterial bioinoculants. Bacterially produced IAA stimulates root development by increasing root length and branching (Feng et al. 2024; Vega-Celedón et al. 2024). Phosphate-solubilizing bacteria convert insoluble organic and inorganic phosphates into forms readily available for plant uptake (Iqbal et al. 2024). Bacterial siderophores may also act as biocontrol agents by restricting iron availability to phytopathogens (Liu et al. 2024). Starch hydrolysis by bacteria facilitates the accumulation of soluble sugars that function as osmolytes, thereby increasing plant drought tolerance (Kaushal and Wani 2016). Finally, polyamines mitigate salinity stress by enhancing the photosynthetic capacity and by strengthening the plant’s antioxidant defense system (ElSayed et al. 2022).

The main goal of this study was to identify potential bacterial bioinoculants by screening bacterial strains for five key PGP-associated traits and to determine whether their functional characteristics and post-inoculation abundance predict plant growth responses across three Poaceae species (*T. intermedium*, *T. aestivum* and *L. perenne*). To the best of our knowledge, this study represents the first functional characterization of bacterial endophytes isolated from *T. intermedium.* We hypothesized that: (i) bacterial strains possessing multiple PGP traits but a narrow spectrum of nutrient utilization would exhibit greater inoculation effectiveness; (ii) the bacterial inoculation outcomes would be host genotype dependent; and (iii) successful bacterial colonization alone would not necessarily predict plant growth promotion. To test our hypotheses, we implemented a multistep experimental approach integrating in vitro PGP trait screening, phenotype profiling using Biolog Phenotype Microarray (PM), plant inoculation experiments, quantification of bacterial abundance by *real-time* quantitative PCR (qPCR), and statistical analyses.

## Materials and methods

### Bacterial strain collection

**Fig. 1:**
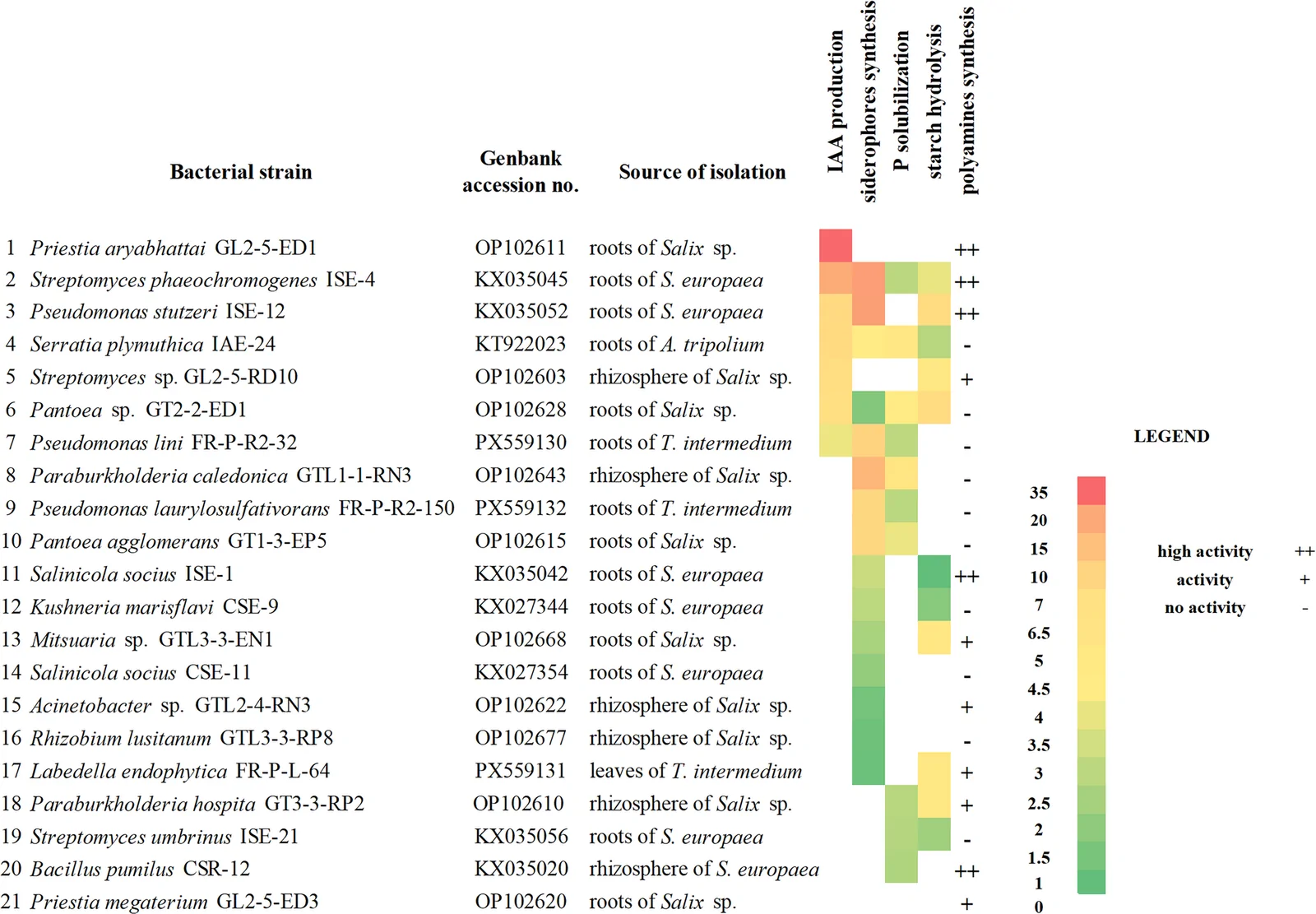
Taxonomic designations, Genbank accession numbers and metabolic traits of 21 endophytic and rhizosphere bacterial strains isolated from four different plant species. The color scale represents the intensity of each activity, along with the respective units or indices used for quantification (siderophore synthesis and starch hydrolysis – W_act_, P solubilization – PSI, IAA production - µg/ml)

A total of 21 bacterial strains were selected for metabolic characterization. The isolates originated from four plant species: *Thinopyrum intermedium* (endophytes from leaves and roots; three strains), *Salicornia europaea* L. (root endophytes and rhizosphere soil; seven strains), *Aster tripolium* L. (root endophyte; one strain), and *Salix* spp. (root endophytes and rhizosphere soil; ten strains). Bacterial strains from *S. europaea*, *Aster tripolium*, and *Salix* spp. were previously isolated and taxonomically identified (Szymańska et al. 2016a, 2016b; Koczorski et al. 2022). The work describing isolation and identification details of strains from *T. intermedium* is currently under preparation. The bacterial and fungal microbiome of *T. intermedium* has been studied in previous works that describe the exact location of the plants (Michl et al. 2025; Deja-Sikora et al. 2026). All strains in this study are obtained from the strain collection of the Department of Microbiology, Nicolaus Copernicus University (Poland). Strain designations and Genbank accession numbers are given in Fig. 1. The bacterial strains associated with halophytes (*S. europaea*, *A. tripolium*) had previously been characterized for metabolic traits, including siderophore production, indole-3-acetic acid (IAA) synthesis, cellulolytic activity, and nitrogen fixation (Szymańska et al. 2016b; 2016a). In contrast, strains isolated from *Salix* spp. had been evaluated only for phosphate-solubilizing activity (Koczorski et al. 2022, 2023). All bacterial strains were revived from glycerol stocks stored at -80 °C, cultured on R2A medium (Difco, BD), and subsequently maintained at 4 °C for further experimentation. The selection of strains from both host (*T. intermedium*) and non-host (*S. europaea* and *Salix* sp.) plant species was intentional to evaluate whether commonly assessed PGP traits can predict plant growth responses across heterogeneous host–strain combinations, including both ecologically matched and mismatched host–microbe associations.

### Screening of strains for plant growth-promoting (PGP) activity

All 21 bacterial strains were screened for the following PGP traits: IAA production, siderophore production, phosphate solubilization, starch hydrolysis, and polyamine synthesis. All assays were performed in triplicate for each bacterial strain.

#### IAA production

IAA production was quantified using the colorimetric method described by Gordon and Weber (1951) with modifications according to Chang et al. (2014). Bacterial strains were grown in 40 ml of Luria–Bertani (LB) broth (Difco, BD) supplemented with 500 µg/ml of L-tryptophan. Cultures were incubated at 26 ± 2 °C with shaking at 120 rpm for four days. Following incubation, cultures were centrifuged at 8 000 rpm for 15 min. An aliquot (2 ml) of the supernatant was mixed with 4 ml of Salkowski’s reagent and incubated in the dark at room temperature for 25 min. The absorbance was measured at 530 nm using a UV-VIS spectrophotometer (UV-1280, Shimadzu). The IAA concentrations were determined from a standard curve prepared with pure IAA standards in the range of 0–50 µg/ml.

#### Siderophore production

Siderophore production was assessed using Chrome Azurol S (CAS) plate assay prepared according to method of Alexander and Zuberer (1991), in which the CAS indicator solution is incorporated into an M9-based agar medium. Bacterial strains were spot-inoculated onto the plates and incubated at 26 ± 2 °C for seven days. Siderophore production was indicated by the formation of an orange halo surrounding the bacterial colony. The siderophore activity was quantified using the coefficient of activity (W_act_) according to the following formula:

$${\mathrm W}_{\mathrm{act}\;}=\;\mathrm{S}_\mathrm{h}^2\;/\;\left(\mathrm{S}_\mathrm{c}\;\times\;\mathrm t\right)$$

where S_h_ represents the diameter of the halo zone (mm), S_c_ is the diameter of the bacterial colony (mm), and *t* is the incubation time in days (Hrynkiewicz et al. 2010).

#### Phosphate solubilization

Phosphate-solubilizing activity was assessed using National Botanical Research Institute’s phosphate growth medium (NBRIP)(Nautiyal 1999) supplemented with tricalcium phosphate (TCP), following the method described by Koczorski et al. (2021). The strains were spot-inoculated on NBRIP agar and incubated at 26 ± 2 °C for seven days. The formation of halo surrounding the bacterial colonies indicated phosphate solubilization. The phosphate solubilization index (PSI) was calculated as the ratio of the total diameter (colony + halo zone) to the colony diameter, according to Premono et al. (1996).

#### Starch hydrolysis

Amylolytic activity was evaluated using starch agar (15 g/L soluble starch, 20 g/L agar). Bacterial strains were streaked on the plates and incubated at 26 ± 2 °C for five days. Following incubation, the plates were flooded with Gram’s iodine solution to detect the presence of residual starch. The formation of clear halo around the bacterial growth indicated starch hydrolysis (Cappuccino and Welsh 2018). The coefficient of activity (W_act_) was calculated.

#### Polyamine synthesis

Polyamine biosynthesis *via* arginine decarboxylation was evaluated using Long Ashton Decarboxylase (LAD) agar supplemented with L-arginine and phenol red, as described by Cloete et al. (2009). The pH of the medium was adjusted to 5.5 prior to sterilization. Strains were spot-inoculated and incubated in the dark at 26 ± 2 °C for five days. A color change of the medium from yellow to red indicated positive arginine decarboxylase activity.

### High-throughput phenotypic profiling using the biolog PM system

Five bacterial strains selected based on the PGP traits (*Labedella endophytica* FR-P-L-64, *Pseudomonas lini* FR-P-R2-32, *Pseudomonas stutzeri* ISE-12, *Streptomyces phaeochromogenes* ISE-4, *Pantoea* sp. GT2-2-ED1) were analyzed for their metabolic profiles using the Biolog Phenotype Microarray (PM) system (Biolog Inc., USA). The assay employed PM5 microplates (Biosynthetic Pathway/Nutrient Stimulation, catalog no. 12141) containing 94 nutrient sources including amino acids, nucleotides, vitamins, cofactors, organic acids, and various carbon- and nitrogen-containing compounds. The PM5 microplate integrates different carbon, nitrogen, sulfur, and phosphorus sources present in other plates (PM1-2, PM3, PM4). To support bacterial growth, standard carbon sources were supplied to inoculating fluid according to the manufacturer’s recommendations. Sodium succinate was used for Gram-negative bacteria and glucose for Gram-positive bacteria. Inoculation was carried out following the protocol of Mackie et al. (2014), with minor modifications. Briefly, bacterial strains were grown in liquid medium for 24–48 h at 26 ± 2 °C and harvested by centrifugation at 5 000 rpm for 5 min. The resulting pellet was resuspended in Biolog inoculating fluid, and the 70% transmittance of the suspension was adjusted using a Biolog turbidimeter (Biolog Inc., USA). Aliquots (100 µL) of the prepared cell suspension were dispensed into each well of the PM5 microplates. All assays were performed in triplicate using the Odin VIII system (Biolog Inc., USA). Absorbance readings at 590 nm were recorded at 20 min intervals throughout a 60 to 96-hour incubation period. Data analysis was conducted using the Odin PM System Software Version 3.1 (Biolog Inc., USA).

### Pot experiment to assess PGP effects of selected bacterial strains on grasses

#### Inoculum preparation

Five bacterial strains (*L. endophytica* FR-P-L-64, *P. lini* FR-P-R2-32, *P. stutzeri* ISE-12, *S. phaeochromogenes* ISE-4, *Pantoea* sp. GT2-2-ED1) were selected based on their PGP traits - IAA production, siderophore synthesis, phosphate solubilization, starch hydrolysis, and polyamine synthesis. Each strain was cultured in LB broth at 26 ± 2 °C for 48 h with shaking, centrifuged at 8 000 rpm for 5 min, and resuspended in sterile distilled water. The optical density of inoculum was adjusted to 0.5 at 600 nm (equivalent to 1.5 × 10^8^ cfu mL^− 1^) using a NanoDrop™ One/OneC spectrophotometer (Thermo Fisher Scientific). Fresh bacterial suspensions were prepared immediately before each inoculation.

#### Experimental design

The experiment included three Poaceae species: *T. intermedium* (Kernza), *L. perenne* (varieties Tetradry and Antonella), and *T. aestivum* (varieties Euforia and Eskapada). Seeds were surface sterilized using 70% ethanol (3 min incubation), 0.5% Triton X-100 (2 min incubation), and 10% hydrogen peroxide (8 min incubation). Then, seeds were rinsed six times with sterile distilled water. Seeds were germinated on moist sterile filter paper in Petri dishes for 4–8 days, depending on species, and subsequently transferred to sterile sand-filled pots (4 × 4 cm). The sand substrate had a particle size range of 0.5–1.4 mm and was sterilized by autoclaving at 121 °C for 20 min. Plants were grown in controlled conditions (20 °C, 9/16 h light/dark) using sodium lighting. Each pot received 1 ml of bacterial suspension with previously adjusted cell concentration at the shoot-root interface at two time points: first at seedling establishment (day 4 or 8), and again on day 24. Sterile Hoagland’s nutrient solution (Hoagland and Arnon 1950) was applied three times per week at a volume of 600 mL per tray. For each plant species, non-inoculated plants served as controls. Each treatment (five bacterial strains and control) consisted of 42 biological replicates. After six weeks of growth, plants were harvested and washed in sterile distilled water to remove substrate particles. Three plant biological replicates per treatment were stored at − 80 °C for chlorophyll content determination and qPCR analysis. The remaining plants were analysed for shoot and root length, fresh weight (FW), and dry weight (DW).

### Quantification of chlorophyll content in plant shoots

Chlorophyll content in shoots was determined following the method of Witham et al. (1971). Frozen plant material (100 mg) was homogenized in liquid nitrogen, and total chlorophyll was extracted using 1 ml of cold 80% acetone. Samples were centrifuged at 5 000 rpm for 5 min, and extraction was repeated until the pellet became colorless. The pooled supernatants were analyzed at 645 and 663 nm against an 80% acetone blank using a UV-VIS spectrophotometer (UV-1280, Shimadzu). Three biological and technical replicates per treatment were analysed. Total chlorophyll concentration was calculated using standard equation:

$$\;\begin{array}{c}Total\;chlorophyll\;=\\(20.2\;\times\;A_{645}\;+\;8.02\;\times\;A_{663})\times\;(V/\;(1000\;\times\;W))\end{array}$$

where, V – final volume of the extract, W – weight of the leaves.

### qPCR-based quantification of bacterial abundance in plant organs

#### Genomic DNA extraction

Genomic DNA was extracted from 100 mg of frozen shoots and roots. Three biological replicates were maintained for each treatment. Samples were homogenized in liquid nitrogen and DNA was isolated using the GeneMATRIX Plant & Fungi DNA Purification Kit (EurX, Poland), following the manufacturer’s protocol. DNA concentration was quantified using the Qubit™ dsDNA HS Assay Kit (Invitrogen™), and samples were stored at − 80 °C until further analysis.

#### Real-time quantitative PCR assay

Strain-specific primers were designed for this study (Table 1). For each bacterial strain, the target sequence was obtained by Sanger sequencing and compared with the closely related reference sequences retrieved from the NCBI RefSeq database. Multiple-sequence alignments were used to identify strain-discriminating regions, and primers generating amplicons shorter than 200 bp were designed within these regions. Primer specificity was evaluated in silico using Primer-BLAST against the 20 most closely related homologous sequences and was further verified experimentally by melting-curve analysis. Quantification of absolute bacterial abundance was performed using a LightCycler 480 system and SYBR Green I Master Kit (Roche). For *L. endophytica* FR-P-L-64, *P. lini* FR-P-R2-32, and *Pantoea* sp. GT2-2-ED1, reactions (10 µl) contained: 5 µl of SYBR Green mix, 3.5 µl of H₂O, 0.25 µl of each primer (10 pmol/µl), and 1 µl of DNA template (5 ng/µl for shoots, 20 ng/µl for roots). For *S. phaeochromogenes* ISE-4 and *P. stutzeri* ISE-12, a 20 µl reaction mix was used: 10.25 µl of SYBR Green mix, 7.25 µl of H₂O, 0.75 µl of each primer (10 pmol/µl), and 1 µl of DNA template (5 ng/µl for shoots, 20 ng/µl for roots).

The thermal cycling conditions consisted of: initial denaturation at 95 °C for 8 min, followed by 40 cycles of denaturation at 95 °C for 10 s, primer-specific annealing (Table 1) for 10 s, and elongation at 72 °C for 20 s. Melting-curve analysis was recorded with a continuous temperature increase from 65 °C to 95 °C. Positive controls (DNA of specific strains) and negative controls (molecular grade water) were included in each run. Each treatment was analyzed using three biological and three technical replicates.

#### Standard curve and data analysis

Absolute quantification of 16 S rRNA copy number was performed using standard curves prepared based on amplicons produced with universal primers 27 F and 1492R (Lane 1991). PCR was conducted using Taq PCR Master Mix (EurX, Poland). The initial denaturation performed at 94 °C for 2 min was followed by 30 amplification cycles (denaturation at 94 °C for 30 s, annealing at 55 °C for 30 s, and extension at 72 °C for 1 min 30 s) and a final extension at 72 °C for 5 min. PCR products were purified with AMPure XP beads (Beckman Coulter), then quantified with a Qubit™ dsDNA HS Assay Kit (Invitrogen™, USA) and Qubit fluorometer (ThermoFisher Scientific, USA). Standard curves were prepared using 5-fold dilutions of amplicons and used to calculate the PCR efficiency for each primer pair. Results were expressed as 16 S rRNA gene copies per ng of total plant DNA.

**Table 1:** Oligonucleotide sequences used in qPCR. The sequences were designed specifically for this research

Primer name	Sequence (5 → 3)	Detected bacteria	Annealing temperature
Streptomyces_F	AATACCGGATAACACTCCCCGAG	*S. phaeochromogenes* ISE-4	58 °C
Streptomyces_R	TCGTCGCCTTGGTGAGCTTC	*S. phaeochromogenes* ISE-4	
Pseudomonas_F	GATGAGTGGAGCTTGCTCCATG	*P. stutzeri* ISE-12	63 °C
Pseudomonas_R	GGCTCATCTGATAGCGTGAGGTC	*P. stutzeri* ISE-12	
Pantoea_F	GACATCCACGGAATTCGGCAGAGATG	*Pantoea* sp. GT2-2-ED1	63 °C
Pantoea_R	CCTTTGAGTTCCCGCCATTACGCGC	*Pantoea* sp. GT2-2-ED1	
L_endophytica_F	CGGAAACGTCTGGAAACAGTCGCCC	*L. endophytica* FR-P-L-64	51 °C
L_endophytica_R	GAGTTGACCCCGGCAGTCTTCC	*L. endophytica* FR-P-L-64	
P_lini_F	GGGAGGAAGGGCAGTTACCTAATACG	*P. lini* FR-P-R2-32	60 °C
P_lini_R	GCTCGTCAGTTTTGAATGCAGTTCCC	*P. lini* FR-P-R2-32	

### Statistical analysis

Raw datasets were checked for outliers and values detected as outliers (*p* < 0.05) were removed from further analysis. Plant growth parameters (length, FW, DW), chlorophyll content, and bacterial abundance within plant organs were analyzed using Statistica software (v. 13.3.721; StatSoft, Poland). The normality of data distribution was checked with Shapiro–Wilk W-test. As the data did not follow a normal distribution, the Kruskal–Wallis test was applied to assess significant differences in the effects of bacterial inoculation compared to control in case of bacterial abundance or between values obtained for each inoculation treatment in case of plant growth parameters and chlorophyll (*p* < 0.05). A linear model (least squares regression) was fitted to assess the effects of endophyte abundance and treatment (plant variety, endophyte inoculation) on plant growth parameters. The log-transformed abundance of endophyte (log10) in roots was included as a continuous predictor. Plant variety was modeled as a categorical fixed effect, with Kernza specified as the reference. The model also included inoculation with endophyte as a categorical predictor (to compare inoculation vs. control variants). Coefficients represent the estimated change in the outcome variable per unit change in the predictor (e.g., log-transformed endophyte abundance). Significance of predictors was assessed using t-test. The analysis was performed in the R software (version 4.2.1) using lme4 package.

The effects of plant variety, bacterial strain, and their interaction on plant growth parameters (length, DW, FW) were evaluated using permutational multivariate analysis of variance (PERMANOVA). Prior to the analysis, the plant growth parameters measured in different units were standardized using z-score normalization. The analysis was conducted on a Euclidean distance matrix in R software (version 4.3.2) using package vegan and the adonis2 function. To verify the PERMANOVA results, the assumption of homogeneity of multivariate dispersions was evaluated using the betadisper function. Principal coordinates analysis (PCoA) was used to visualize clustering of samples according to plant variety and bacterial strain.

## Results

### Functional characterization and selection of PGP bacterial candidates

The metabolic properties of the bacterial strains are summarized in Fig. 1 and the corresponding numerical values are provided in Supplementary information Table S1. The sequence in which the PGP traits are presented reflects their potential contribution to plant growth (IAA production), nutrient assimilation (siderophore production, phosphate solubilization), and stress mitigation (starch hydrolysis, polyamine synthesis). In this study, we prioritized not only the presence of individual PGP activities displayed by bacterial strains originating from different sources (host plant and plant organ) but also on their combine occurrence of multiple traits to identify strains best-suited as PGP bioinoculants. The strains exhibiting IAA production together with at least one additional trait associated with nutrient assimilation or stress mitigation were preferentially selected for further analysis.

IAA production was detected in only 7 out of the 21 bacterial strains tested. The highest levels of IAA were observed for *S. phaeochromogenes* ISE-4 (20 µg/ml) and *Priestia aryabhattai* GL2-5-ED1 (35 µg/ml), while the lowest level of IAA production was reported for *P. lini* FR-P-R2-32 (3 µg/ml). Siderophore synthesis was observed in 16 out of the 21 bacterial strains tested. The most efficient siderophore producers (W_act_ = 15–20) included *P. stutzeri* ISE-12, *S. phaeochromogenes* ISE-4, and *Paraburkholderia caledonica* GTL1-1-RN3. In contrast, weak siderophores production (below W_act_ = 2) was found for *Acinetobacter* sp. GTL2-4-RN3, *Rhizobium lusitanum* GTL3-3-RP8, *Pantoea* sp. GT2-2-ED1, and *L. endophytica* FR-P-L-64. Only 10 out of the 21 bacterial strains tested demonstrated the ability to solubilize phosphate. Bacterial strains that displayed high phosphate solubilization capacity (PSI = 4–5) included *Serratia plymuthica* IAE-24 and *P. caledonica* GTL1-1-RN3. The lowest value (PSI = 2) of phosphate solubilization was observed for *Bacillus pumilus* CSR-12. Starch was hydrolyzed by 11 strains, and most efficiently (W_act_ = 7) by *P. stutzeri* ISE-12 and *Pantoea* sp. GT2-2-ED1, while *Salinicola socius* ISE-1 demonstrated the lowest amylolytic activity (W_act_ = 1). The strongest ability to synthesize polyamines was characteristic for *P. aryabhattai* GL2-5-ED1, *S. phaeochromogenes* ISE-4, *P. stutzeri* ISE-12, *S. socius* ISE-1, and *B. pumilus* CSR-12. Overall, endophytic bacteria displayed higher PGP activity than rhizosphere strains.

Based on their prominent PGP profiles, four strains isolated from roots were selected for further analyses, including phenotypic profiling and plant inoculation experiments: *P. stutzeri* ISE-12 and *S. phaeochromogenes* ISE-4 (both isolated from *S. europaea*), *Pantoea* sp. GT2-2-ED1 (isolated from *Salix* spp.), and *P. lini* FR-P-R2-32 (​isolated from *T*. *intermedium*). Additionally, *L. endophytica* FR-P-L-64 (also isolated from *T. intermedium*) was included in the selection as the only endophytic strain derived from leaves. Compared to selected root endophytes, this strain exhibited low metabolic potential, characterized by weak ability to produce siderophores, starch hydrolysis, and polyamine synthesis​. 

### Nutrients utilization by bacterial strains

**Fig. 2:**
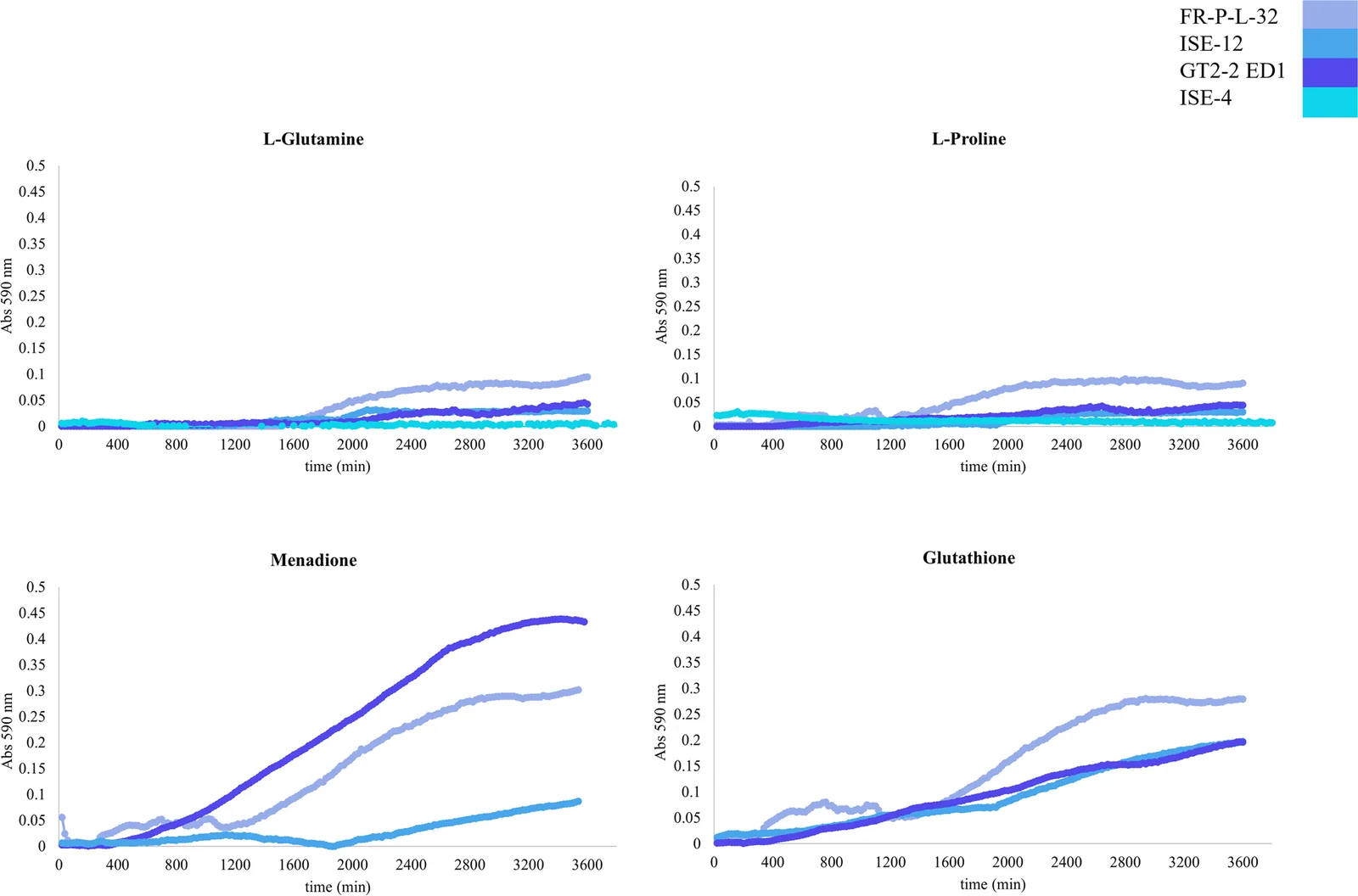
Line graphs show utilization of four selected substrates determined with Biolog PM5 assay by four bacterial strains (*P. lini* FR-P-R2-32, *P. stutzeri* ISE-12, *Pantoea* sp. GT2-2-ED1, *S. phaeochromogenes* ISE-4) at 60 h incubation

The Biolog PM5 assay was used to characterize nutrient utilization patterns and substrate-dependent growth responses of five bacterial strains. *L. endophytica* FR-P-L-64 did not exhibit growth or respiration under assay conditions and therefore was excluded from further phenotype analysis. Distinct phenotypic profiles were observed among the four bacterial strains across 94 nutrient sources (Fig.S1, Table S2). The highest nutrient utilization spectrum was exhibited by *P. lini* FR-P-R2-32, while, in contrast, *S. phaeochromogenes* ISE-4 metabolized only 10 substrate sources including amino acids (4), nucleotides (2), vitamins and cofactors (1), organic acids (1), and lipids and detergents (2). Substrate selectivity depended on their biochemical category and differed between bacterial strains. The growth of *P. lini* FR-P-R2-32, *P. stutzeri* ISE-12, and *Pantoea* sp. GT2-2-ED1 was stimulated at different levels by compounds belonging to amino acids and nucleotides. In contrast, these three bacterial strains showed comparable growth patterns with different energy sources. Vitamins and cofactors were utilized similarly by both *Pseudomonas* strains, while *Pantoea* sp. GT2-2-ED1 was more selective in this category of nutrients. Notably, the growth of *P. stutzeri* ISE-12 did not differ in the presence of lipids and detergents. To examine metabolites relevant to plant–microbe interactions, we focused on selected compounds (Fig. 2). Amino acids L-glutamine and L-proline were utilized by four bacterial strains at different levels. *S. phaeochromogenes* ISE-4 exhibited minimal growth in the presence of amino acids, while *P. stutzeri* ISE-12 and *Pantoea* sp. GT2-2-ED1 demonstrated weak utilization. In contrast, *P. lini* FR-P-R2-32 showed the highest metabolic response to both substrates. Menadione (vitamin K3) and glutathione stimulated the growth of three bacterial strains. The highest level of menadione utilization was observed for *Pantoea* sp. GT2-2-ED1, and glutathione was found to facilitate the growth of *P. lini* FR-P-R2-32 most efficiently. *P. stutzeri* ISE-12 and *Pantoea* sp. GT2-2-ED1 exhibited similar glutathione metabolism.

### Plant growth responses to bacterial inoculation

**Fig. 3:**
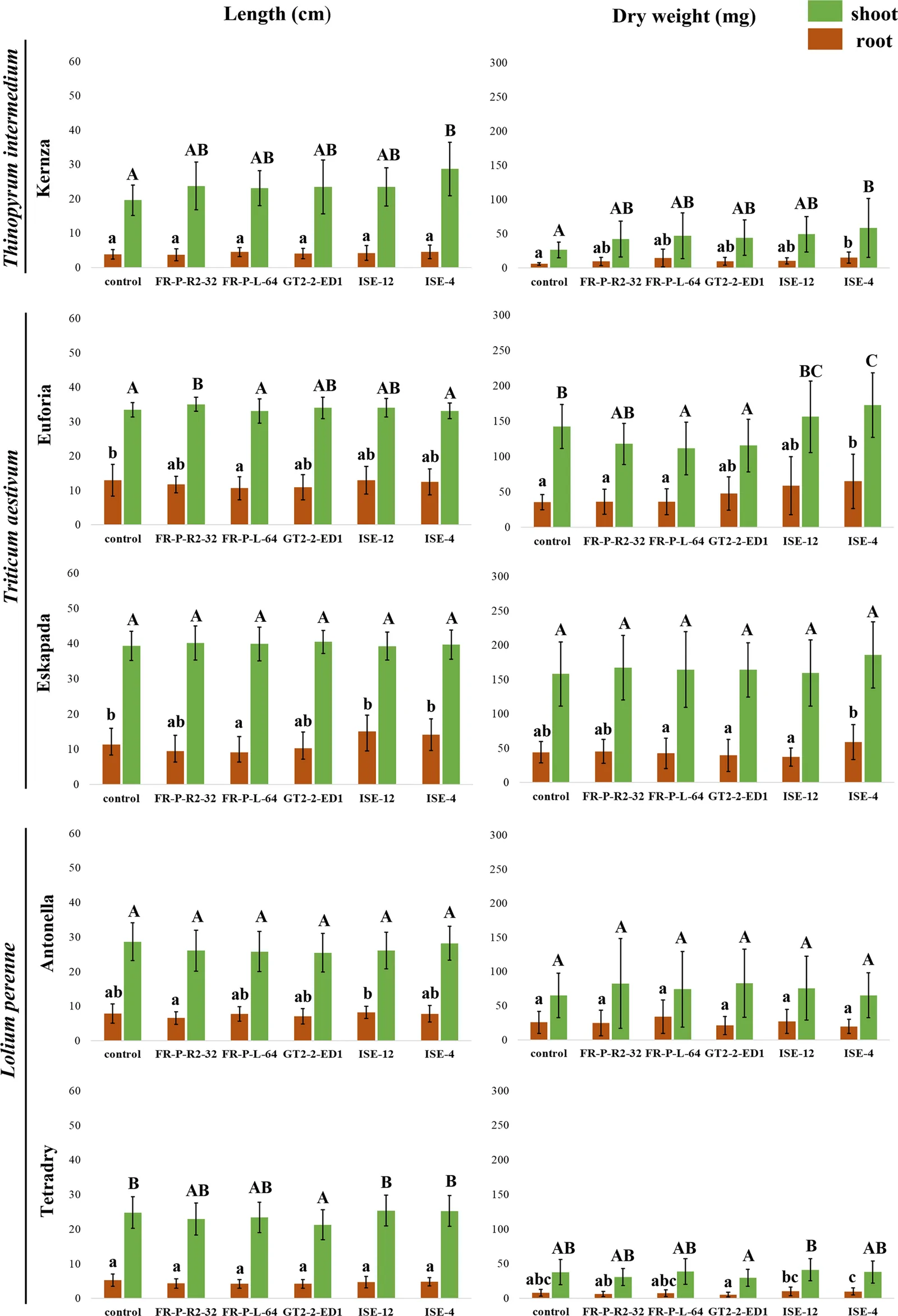
Growth of five plant varieties inoculated with different bacterial strains. The bars indicate mean ± SD (*n* = 32–36). Different letters denote significant differences (*p* < 0.05, Kruskal-Wallis test) between tested treatments. Abbreviations: FR-P-R2-32 - *P. lini* FR-P-R2-32, FR-P-L-64 - *L. endophytica* FR-P-L-64, GT2-2-ED1 - *Pantoea* sp. GT2-2-ED1, ISE-12 - *P. stutzeri* ISE-12, ISE-4 - *S. phaeochromogenes* ISE-4

No significant growth responses were observed in the *L. perenne* variety Antonella following bacterial inoculation compared to non-inoculated control plants. The plant growth parameters (length and DW of roots and shoots) of the remaining four plant varieties (Kernza, Eskapada, Euforia, Tetradry) varied depending on the inoculated bacterial strain (Fig. 3, Fig. S2).

In Kernza, inoculation with *S. phaeochromogenes* ISE-4 significantly increased shoot length, as well as shoot and root DW. In *T. aestivum* variety Euforia, *S. phaeochromogenes* ISE-4 significantly increased both root and shoot DW, whereas *P. lini* FR-P-R2-32 significantly increased the shoot length. In contrast, inoculation with strain *Pantoea* sp. GT2-2-ED1 resulted in a significant decrease in shoot DW in *T. aestivum* variety Euforia and shoot length in *L. perenne* variety Tetradry. *L. endophytica* FR-P-L-64 negatively affected both *T. aestivum* varieties (Eskapada and Euforia) by reducing root length and shoot DW.

Inoculation with *P. stutzeri* ISE-12 did not significantly affect any measured growth parameter in the five tested Poaceae varieties compared with control plants. Two strains exhibiting multiple PGP traits, *S. phaeochromogenes* ISE-4 and *P. lini* FR-P-R2-32, improved the plant growth; however, their beneficial effect was limited to specific host genotypes. Conversely, strains displaying PGP-associated characteristics, such as *Pantoea* sp. GT2-2-ED1 and *L. endophytica* FR-P-L-64, produced negative growth responses in particular host–strain combinations.

PERMANOVA analysis revealed that the multivariate profile of plant growth parameters was significantly affected by plant variety (R² =0.502, *p* = 0.001), bacterial strain (R² = 0.029, *p* = 0.001), and the variety × strain interaction (R² = 0.066, *p* = 0.001). Plant variety explained 50.2% of the observed variation in plant growth parameters, followed by variety x strain interaction (6.6%) and strain (2.9%) (Table S3). PCoA supported these findings, showing clear clustering of samples according to plant variety, while separation based on bacterial strains was less evident (Fig. S3). The test for homogeneity of multivariate dispersions was also significant (*p* = 0.001), indicating differences in within-group variability. Therefore, PERMANOVA results should be interpreted cautiously, as part of the observed differences may reflect variation in group dispersion rather than differences in group centroids alone.

### Chlorophyll content in plants after inoculation

**Fig. 4:**
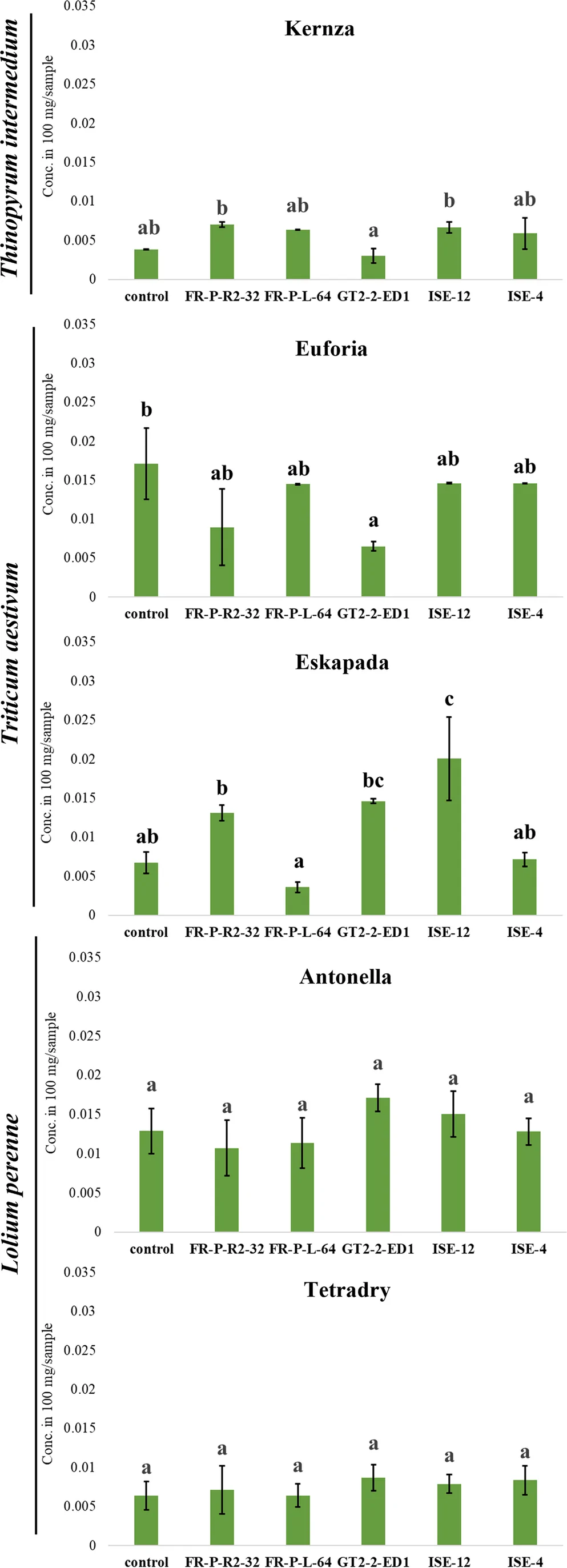
Chlorophyll content in shoots of five plant varieties inoculated with different bacterial strains. The bars indicate mean ± SD (*n* = 3). Different letters denote significant differences (*p* < 0.05, Kruskal-Wallis test) between tested treatments. Abbreviations: FR-P-R2-33 -*P. lini* FR-P-R2-32, FR-P-L-64 - *L. endophytica* FR-P-L-64, GT2-2-ED1 - *Pantoea* sp. GT2-2-ED1, ISE-12 - *P. stutzeri* ISE-12, ISE-4 - *S. phaeochromogenes* ISE-4

Inoculation of the two *L. perenne* varieties (Antonella and Tetradry) and Kernza with the five selected bacterial strains did not significantly affect chlorophyll content compared with the respective controls. Among the five tested Poaceae varieties, only the two *T. aestivum* varieties exhibited significant responses to bacterial inoculation (Fig. 4). In *T. aestivum* variety Eskapada, inoculation with *P. stutzeri* ISE-12 significantly increased chlorophyll concentration. Conversely, a decrease in chlorophyll level was noted after inoculation of *T. aestivum* variety Euforia with *Pantoea* sp. GT2-2-ED1. Overall, despite the presence of multiple PGP-associated traits, most bacterial strains did not enhance chlorophyll accumulation across the tested Poaceae hosts, indicating that their effects on photosynthetic parameters were strongly dependent on the plant genotype–strain combination.

### Quantitative analysis of bacterial abundance in plant organs with qPCR

**Fig. 5:**
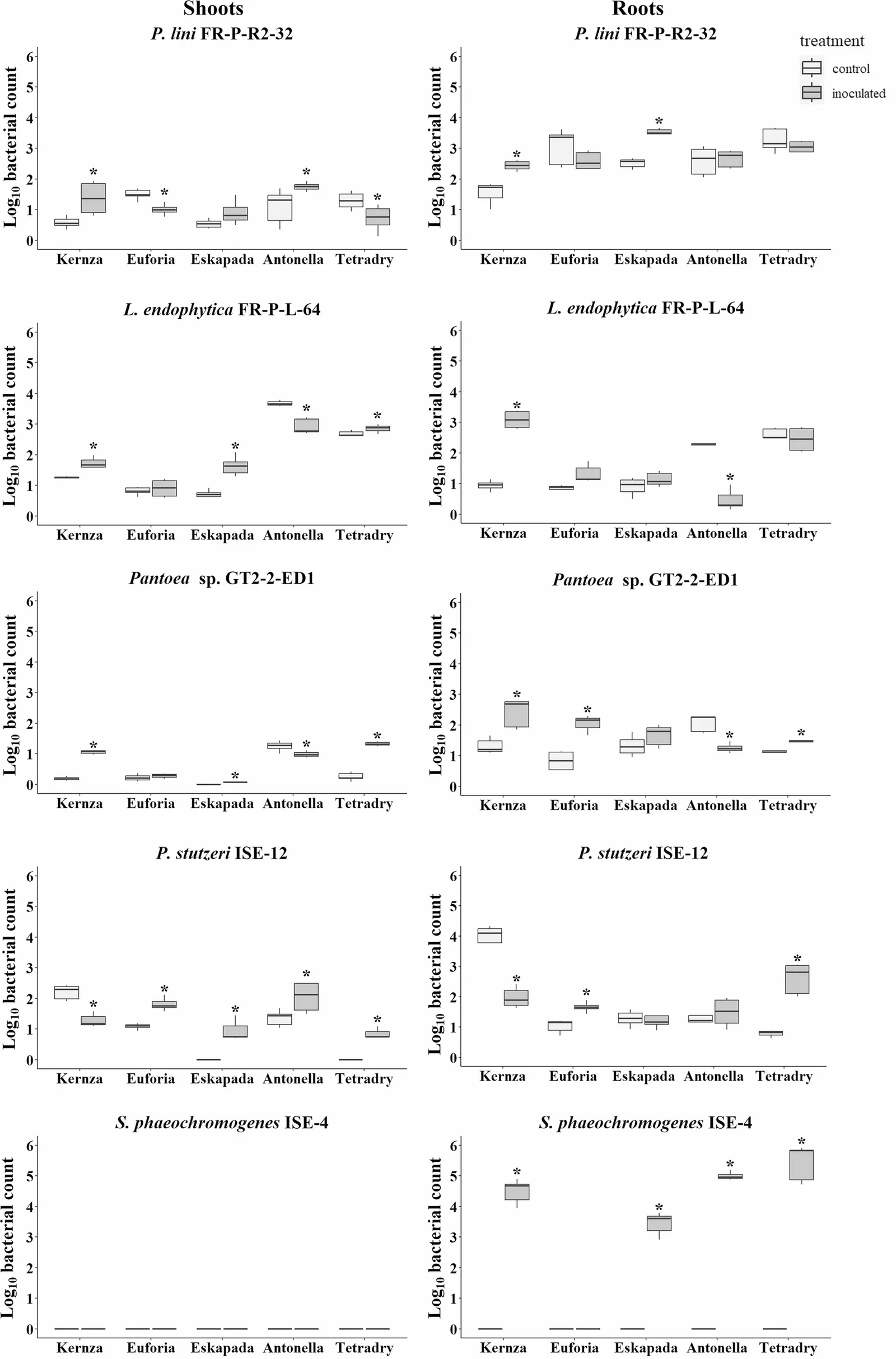
Log-transformed absolute bacterial abundance (log10) in shoots and roots of *T. intermedium* (Kernza), *T. aestivum* (Euforia, Eskapada), and *L. perenne* (Antonella, Tetradry). The box plots represent medians and interquartile ranges. Significant differences (*p* < 0.05, Kruskal-Wallis test) between control and inoculated treatments are denoted by (*)

The qPCR results revealed distinct differences in the distribution and abundance of bacterial endophytes among five Poaceae varieties (Fig. 5, Fig. S4, Fig. S5). Based on these observations, we defined three colonization patterns to facilitate the interpretation of the results: systemic colonization (characterized by an increase of bacterial abundance in shoots and roots after inoculation), limited colonization (defined as no change or decrease of bacterial abundance in shoots and roots after inoculation), and organ-specific colonization (defined as changes in bacterial abundance after inoculation depended on the plant organ). In Kernza, systemic colonization by *P. lini* FR-P-R2-32, *L. endophytica* FR-P-L-64, and *Pantoea* sp. GT2-2-ED1 was characteristic, while *P. stutzeri* ISE-12 demonstrated limited colonization. The organ-specific colonization by *Pantoea* sp. GT2-2-ED1 and systemic colonization by *P. stutzeri* ISE-12 were observed in Euforia. In contrast, *P. lini* FR-P-R2-32 and *L. endophytica* FR-P-L-64 showed limited colonization. Notably, the colonization of Eskapada was organ-specific for all bacterial endophytes. In Antonella, the limited colonization demonstrated *L. endophytica* FR-P-L-64 and *Pantoea* sp. GT2-2-ED1. Simultaneously, colonization by *P. lini* FR-P-R2-32 and *P. stutzeri* ISE-12 was organ-specific in Antonella. The systemic colonization by *Pantoea* sp. GT2-2-ED1 and *P. stutzeri* ISE-12 was found in Tetradry, while colonization by *P. lini* FR-P-R2-32 and *L. endophytica* FR-P-L-64 was organ-specific in this plant variety. Finally, the colonization by *S. phaeochromogenes* ISE-4 in Kernza, Eskapada, Antonella, and Tetradry was organ-specific, and this strain was detected only after inoculation.

### Evaluation of the effect of inoculation on the bacterial abundance and dry weight in Poaceae varieties using statistical models

**Fig. 6:**
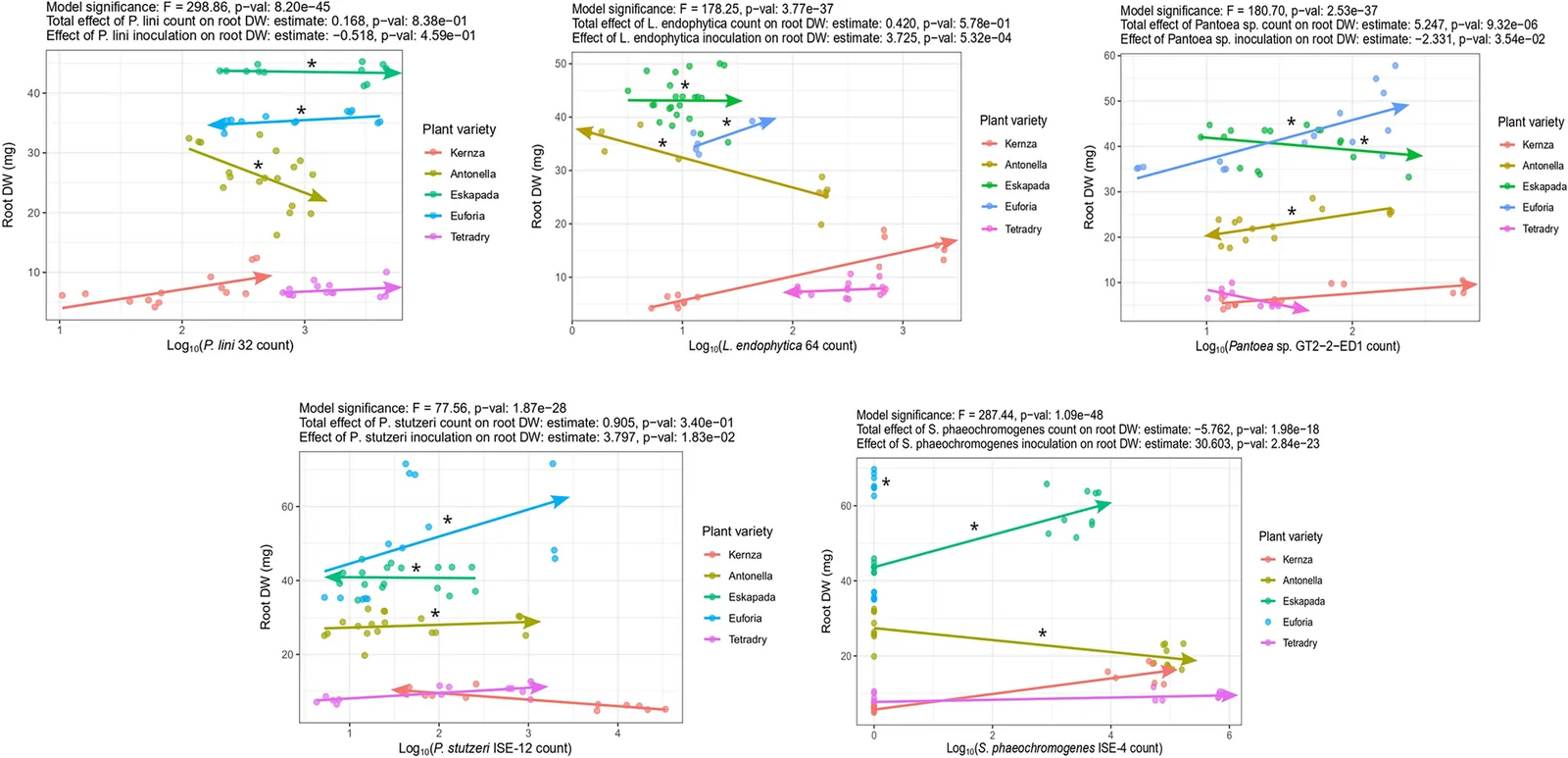
Linear regression models showing the effects of endophyte abundance and treatment (plant variety, endophyte inoculation) on root dry weight (DW). Significant differences (*p* < 0.05, t-test) between plant varieties (Antonella, Eskapada, Euforia, Tetradry) and reference (Kernza) are marked by (*)

**Fig. 7:**
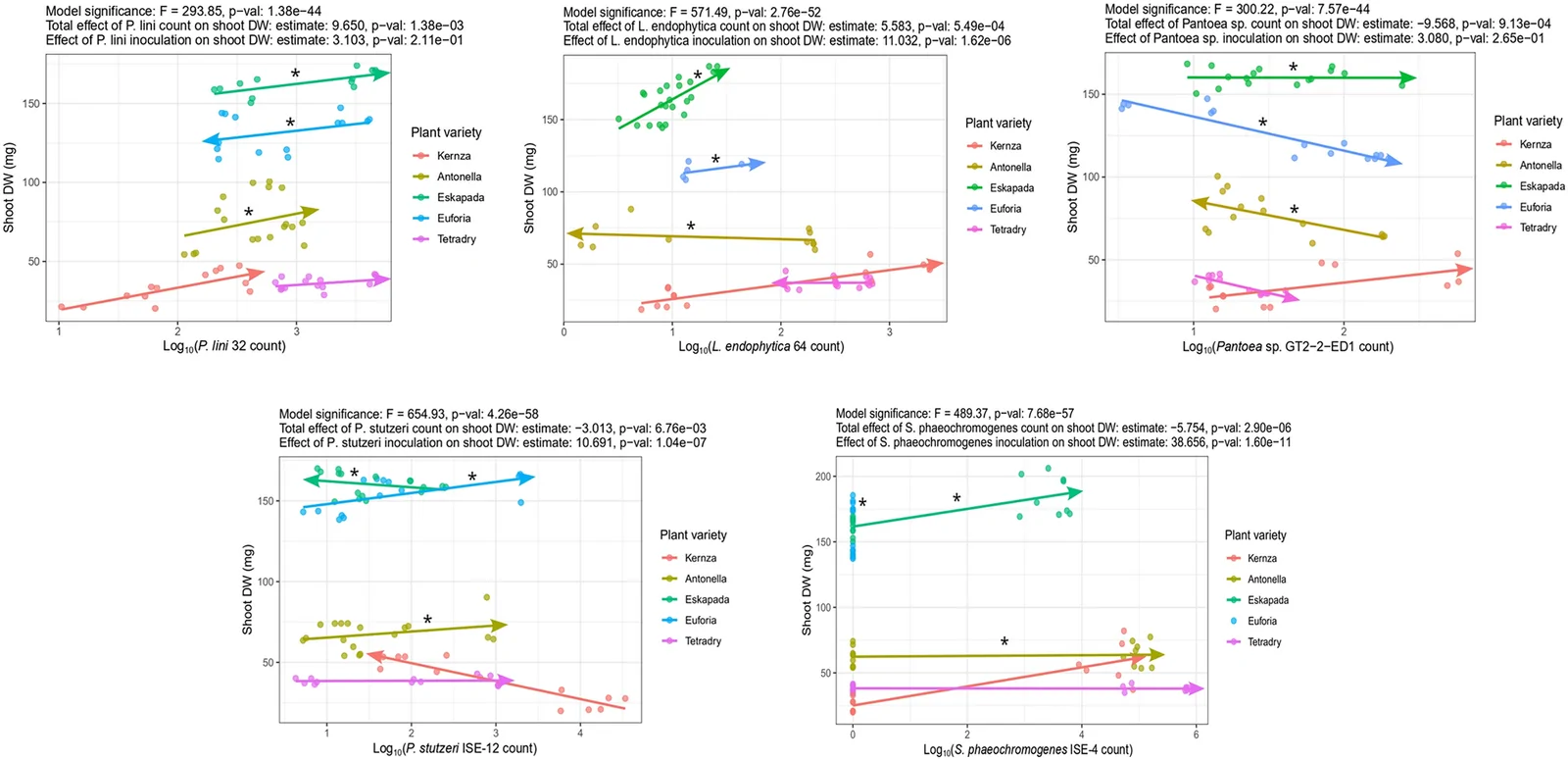
Linear regression models showing the effects of endophyte abundance and treatment (plant variety, endophyte inoculation) on shoot dry weight (DW). Significant differences (*p* < 0.05, t-test) between plant varieties (Antonella, Eskapada, Euforia, Tetradry) and reference (Kernza) are marked by (*)

Significant differences were observed between Kernza (reference) and three plant varieties (Euforia, Eskapada, and Antonella) for shoot and root DW across inoculation treatments. In contrast, Tetradry showed similar DW to Kernza after inoculation with bacterial endophytes (Figs. 6 and 7). The effect of bacterial abundance on DW was strongly dependent on plant variety. Associations were descriptively classified as positive when bacterial abundance increased shoot and root DW after inoculation, and negative when bacterial abundance decreased shoot and root DW after inoculation. An opposite-direction association was defined as a positive association between root bacterial abundance and DW in one plant organ, and a negative association in the other. Positive associations were demonstrated after inoculation with all five bacterial endophytes in Kernza. Bacterial abundance and DW of Tetradry showed an opposite-direction association after inoculation with *L. endophytica* FR-P-L-64 and *Pantoea* sp. GT2-2-ED1. Positive associations were identified in Tetradry with *P. lini* FR-P-R2-32 and *P. stutzeri* ISE-12 inoculation. In Antonella, positive associations were observed after inoculation with *L. endophytica* FR-P-L-64 and *P. stutzeri* ISE-12. Opposite-direction associations in Antonella were identified after inoculation with *P. lini* FR-P-R2-32, *Pantoea* sp. GT2-2-ED1, and *S. phaeochromogenes* ISE-4. Notably, the positive association in Eskapada was observed only after inoculation with *S. phaeochromogenes* ISE-4, whereas in the other inoculation treatments, an opposite-direction association was observed. The positive associations were indicated in Euforia after inoculation with *L. endophytica* FR-P-L-64 and *P. stutzeri* ISE-12. The association was negative after inoculation of Euforia with *P. lini* FR-P-R2-32, while an opposite-direction association was observed after inoculation with *Pantoea* sp. GT2-2-ED1. Since *S. phaeochromogenes* ISE-4 was not detected after inoculation in Euforia, the association between bacterial abundance and DW was not analyzed. The associations between root bacterial abundance and other plant growth parameters (i.e., length and FW) are presented in the Supporting information (Fig. S6, S7, S8, and S9).

## Discussion

### PGP properties and nutrient utilization patterns were insufficient to predict inoculation outcomes across heterogeneous host–strain combinations

This work evaluated the PGP potential of endophytic bacteria previously isolated from perennial crop Kernza (unpublished data) and from other host plants including *Salicornia europaea* L., *Aster tripolium* L., and *Salix* spp. (Szymańska et al. 2016a, 2016b; Koczorski et al. 2022). The selection of bacterial bioinoculants was based on five key PGP traits commonly associated with improved plant performance and stress resilience, including IAA production, phosphate solubilization, siderophore synthesis, starch hydrolysis, and polyamine synthesis. The first hypothesis in our work proposed that bacterial strains exhibiting multiple PGP traits combined with narrow spectrum of nutrient utilization would show greater inoculation effectiveness.

This hypothesis was based on prior findings demonstrating that bacterial strains with multiple PGP traits contributed to increased plant performance. *P. stutzeri* expressing a wide range of PGP traits, e.g., IAA and siderophores synthesis, nitrogen fixation and pectinase production, enhanced crop growth (Saha et al. 2016; Geries and Elsadany 2021). Robust PGP activity, including phosphate solubilization, phytohormone production, siderophore synthesis, and hydrogen cyanide (HCN) production, and its effective application in PGP was reported for the strain *P. lini* (Maheshwari et al. 2022). Likewise, *Pantoea* strains were characterized as highly efficient phosphate solubilizing bacteria and wheat growth promoters (Prasad et al. 2022), whereas endophytic *Streptomyces* strains were positively tested for PGP traits and evaluated for growth-promoting benefits in *Arabidopsis thaliana* (Worsley et al. 2020). In this work, the results showed that inoculation outcomes cannot be predicted solely based on PGP properties across heterogeneous host-strain combinations. *P. stutzeri* ISE-12, *P. lini* FR-P-R2-32, *S. phaeochromogenes* ISE-4, *L. endophytica* FR-P-L-64, and *Pantoea* sp. GT2-2-ED1 were characterized by PGP properties but demonstrated different effects on plant growth.

Biolog PM profiling revealed substantial differences in nutrient utilization patterns among the investigated bacterial strains, highlighting their functional diversity and potential ecological specialization. Biolog PM microplates have been widely used to characterize metabolic versatility in bacteria and to infer their potential ecological functions (Li et al. 2017; Musialowski et al. 2023; Vitali et al. 2025; Wang et al. 2025). However, *L. endophytica* FRPL 64 did not show growth under the Biolog assay conditions in our study, and its nutrient utilization patterns could therefore not be assessed. Similar lack of growth under Biolog assay conditions has been reported for other bacterial strains (Vick et al. 2019; Akindolire et al. 2025). Such a lack of growth may result from limitations of the assay medium, including the suitability of the supplied carbon sources, or from specific nutritional requirements of the strain that are not met under the Biolog assay conditions (Viti et al. 2015). Thus, the absence of bacterial growth should not necessarily be interpreted as a lack of metabolic potential and requires further investigation. A previous study using the Biolog PM plates with bacterial strain *Paenibacillus polymyxa* analyzed its various carbon, nitrogen, phosphate, and sulfur utilization patterns, and confirmed the presence of genes associated with the metabolism of these nutrients (Vitali et al. 2025).​ *Pseudomonas* species have been previously reported as efficient utilizers of different nitrogen, carbon, phosphorus, and sulfur sources using Biolog PM plates (Li et al. 2017; Musialowski et al. 2023). In this study, *P. lini* FR-P-R2-32 exhibited the broadest metabolic utilization of amino acids, nucleotides, and redox-active compounds, while *S. phaeochromogenes* ISE-4 utilized a narrow substrate range, suggesting a more specialized metabolic strategy. Notably, metabolic modeling of microbial communities showed that narrower resource utilization correlates with increased metabolic interaction potential and plant growth promotion (Wang et al. 2025). Bacteria from cooperative communities are characterized by fewer metabolic genes and stronger metabolic interactions that facilitate their ecological adaptability (Machado et al. 2021). Consistent with this concept, the results showed that *S. phaeochromogenes* ISE-4 as a narrow-spectrum resource-utilizing strain promoted plant growth, likely due to its metabolic cooperation with the host microbiome. However, the plant growth promoting effects were observed only in two plant genotypes, further confirming that nutrient utilization capacities, similar to PGP traits, did not predict the effectiveness of inoculation.

Further, focusing on specific compounds, potential functions of tested strains in plant-microbe interaction and their ecological specialization were evaluated. Amino acids are known to function as signaling molecules, defense compounds, and nutrients to shape plant-microbe interaction (Moormann et al. 2022). Therefore, differences in amino acids metabolism may influence bacterial adaptability to plant-associated niches and might be crucial for establishing symbiotic interaction. The poor utilization of L-proline and L-glutamine by tested strains suggests limited preference for certain amino acids as carbon and nitrogen sources. Utilization of glutathione and menadione by the three tested endophytes may indicate their roles in modulating oxidative stress and pathogen defense *in planta*. Glutathione plays a role in bacterial competition and modulation of plant-pathogen interaction, especially under sulfur deficiency (Mukherjee et al. 2025). Menadione induces oxidative stress and promotes plant defense response (Aswani et al. 2019). Therefore, bacterial metabolism of menadione suggests their ability to maintain cellular redox homeostasis by modulating its concentration during plant stress response.

### Host genotype determines plant response to inoculation

The plant genotype has been shown to play an important role in determining interactions between PGP bacteria and different crop genotypes like rice, wheat, and potato cultivars with PGPB (Andreote et al. 2010; García de Salamone et al. 2012; Kazi et al. 2016). The second hypothesis of this study proposed that the bacterial inoculation outcomes would be host genotype dependent. The results strongly support this hypothesis, demonstrating that plant responses to bacterial inoculations were highly species- and variety-specific. We observed that inoculation with the bacterial endophytes stimulated, reduced, or did not affect plant growth depending on plant genotype. Among the five tested strains, only *S. phaeochromogenes* ISE-4 consistently promoted plant growth in two genotypes: Kernza and wheat cultivar Euforia. *P. lini* FR-P-R2-32 promoted shoot length in Euforia but did not produce consistent effects across all genotypes, highlighting that the metabolic versatility alone does not ensure universal efficacy. Importantly, *Pantoea* sp. GT2-2-ED1 frequently suppressed plant growth in certain hosts, despite exhibiting PGP traits in vitro. PERMANOVA analysis indicated that plant variety explained the largest proportion of multivariate variation in plant growth responses. However, because significant differences in within-group variability were detected, these results should be interpreted cautiously. Previous studies reported genotype-specific effects of *P. stutzeri* ISE-12, where growth stimulation occurred in barley but not in lettuce or sunflower without salt and under different NaCl concentrations (Szymańska et al. 2022). In this study, *P. stutzeri* did not affect growth across Poaceae genotypes, further confirming that even metabolically competent strains may fail to induce consistent benefits. Analogous results were reported in maize, where rhizobacterial inoculation yielded differential growth and nitrogen-use efficiency across genotypes (Foster et al. 2025) and in wheat, where *Streptomyces* strains induced cultivar-dependent physiological and molecular responses (Akbari et al. 2020).

The variation in plant genotype response to inoculation is due to the production of different types of root exudates (Sasse et al. 2018), which directly affects the ability of microbes to colonize a specific plant genotype (Kuzmicheva et al. 2017). In particular, plant genotype is known to shape bacterial composition in roots, rhizosphere, and phylosphere (Aira et al. 2010; Bodenhausen et al. 2014; Marques et al. 2014) by production of root exudates (Sasse et al. 2018). The root exudate composition and concentrations of selected exudates (sugars and jasmonic acid) were proposed in studies with maize genotypes to explain the different effects of bacterial inoculation (Foster et al. 2025). Host genetics has also been shown to influence plant responsiveness to inoculation (Wintermans et al. 2016; Vidotti et al. 2019). The genetic variation of *Arabidopsis thaliana* was reported to affect its interaction with *Pseudomonas simiae* caused by the plant genes regulating photosynthetic capacity, lateral root formation, shoot fresh weight, and polyamines biosynthesis (Wintermans et al. 2016). In another study, the response of maize genotypes to bacterial inoculation was controlled by the plant genes associated with the defense response to pathogens, modulation of phytohormones, and plant’s immune system (Vidotti et al. 2019).

In our experiment, increased chlorophyll content was detected only in wheat variety Eskapada inoculated with *P. stutzeri* ISE-12. These findings indicate that bacterial effects on photosynthetic performance are also dependent on the host genotype–strain combination. The genotype-specific effects of bacterial inoculation on chlorophyll content have been shown in previous studies investigating the effects of inoculation in different wheat cultivars (Kazi et al. 2016; Akbari et al. 2020).

### Colonization alone does not reflect the inoculation effectiveness

Previous studies have applied statistical models to investigate tomato yield under different microbial inoculation treatments (Sanam et al. 2022) and linked post-inoculation shifts in microbial abundance and richness with plant growth responses such as grain yield, biomass, and nutrient content (Sahib et al. 2020; Chen et al. 2021; Feng et al. 2023). Building on these approaches, we used regression models to test our third research hypothesis, that the successful bacterial colonization alone would not necessarily predict plant growth promotion. The linear regression analysis addresses two key questions: (i) whether the abundance of endophytic bacteria in roots affects specific plant growth parameters; (ii) whether Kernza growth parameters differed from those of other plant varieties in response to inoculation with the tested strains. The statistical models showed positive, negative, and opposite-direction associations between root bacterial abundance and DW depending on plant genotype. Across all regression models, Kernza served as a reference, revealing significant differences in response to bacterial inoculation between this perennial cereal and the other tested Poaceae varieties. For example, increased abundance of *S. phaeochromogenes* ISE-4 in Kernza and Eskapada after inoculation corresponded with improved DW, indicating inoculation effectiveness. In contrast, we observed that bacterial abundance decreased in some inoculation treatments, leading to the reduction of DW in specific plant genotypes.

These findings highlight that the successful colonization alone does not guarantee growth promotion, and that inoculation effectiveness depends highly on the host genotype. This is in line with a previous study that demonstrated different responses of *Brachypodium distachyon* genotypes to inoculation with PGP rhizobacteria. A positive growth response was observed only for few genotypes, despite good root colonization detected for most of the tested genotypes (Amaral et al. 2016). Similarly, *Pseudomonas* strain was shown to reduce maize biomass even when well-established in the root system (Mosimann et al. 2017) and the plant growth promotion of sorghum after successful colonization by the bacterial Syncoms was affected by the host genotype (Chai et al. 2021). To understand variation in plant responses to colonization, several studies evaluated plant immune and molecular patterns (Pršić and Ongena 2020; Tzipilevich et al. 2021; Koprivova et al. 2025). A research on the response of *Arabidopsis* to the PGP bacterial strain and plant pathogen showed that the plant can distinguish between beneficial and detrimental bacteria by involving transcriptome, proteome, and metabolome regulatory mechanisms (Koprivova et al. 2025). However, the colonization of maize and wheat roots with the PGP bacteria resulted in induction of genes or proteins involved in plant pathogen defense (do Amaral et al. 2014; Camilios-Neto et al. 2014). Importantly, it was observed in a previous study that inoculation with auxin producing *Bacillus velezensis* triggered the plant immune system and led to stress response by ROS release (Tzipilevich et al. 2021). The auxin produced by the bacteria antagonized the plant immune reaction and promoted a beneficial interaction with the plant. This phenomenon aligns with the concept of balanced antagonism, meaning an equilibrium between endophyte virulence and plant defense. Its disturbance can lead to development of disease, stress, and weakening of the host defense (Schulz et al. 2019; Zhao et al. 2024). In this study, inoculation with specific strains could have resulted in the disturbance of the host equilibrium and activation of the plant immune response, which in consequence suppressed the colonization and reduced plant growth performance.

The quantitative approach used in this study has some limitations. Since 16 S rRNA gene copy numbers vary among bacterial genomes, gene abundance cannot be directly converted into bacterial cell numbers. Because genome and copy-number information was unavailable for the strains studied, the results should be interpreted as bacterial 16 S rRNA gene abundance normalized to plant DNA, rather than absolute bacterial cell numbers. Additionally, the cross-amplification testing against closely related non-target strains was not experimentally performed. Although sequence comparisons and melting-curve analysis supported the primer specificity, amplification of closely related endophytic bacteria cannot be completely excluded.

### Practical limitations and translational challenges for the application of bacterial bioinoculants

An important limitation of the present study is the use of sterile quartz sand supplemented with Hoagland solution. This simplified system was intentionally chosen to reduce interference from indigenous microbial communities and investigate the effects of the inoculated strains; however, it does not reproduce the physicochemical complexity of agricultural soil or competitive interactions with the native microbiome. Consequently, the inoculation responses observed here should be considered as a controlled proof-of-concept rather than direct evidence of field efficacy.

Although the obtained results suggest that *S. phaeochromogenes* ISE-4 has potential as a bioinoculant for improving growth of specific Poaceae genotypes under controlled pot conditions, several practical limitations should be considered before its application in sustainable agriculture. The performance of PGP endophytes under controlled experimental conditions may differ substantially from the complex field environments. In the field, factors such as soil type, climate, native microbial communities, and agricultural management practices can influence bacterial colonization, persistence, and efficacy. Furthermore, the observed strain- and host genotype-specific responses in our work indicate that bacterial inoculants effective for one Poaceae species or variety may not produce comparable benefits in another, highlighting the importance of evaluating host–microbe compatibility before large-scale application. Additional translational challenges may include the development of stable and cost-effective bioinoculant formulations, optimization of inoculation method, and assessment of long-term effects on plant performance and native soil microbial communities. Future research should therefore focus on field-based validation under diverse environmental conditions, optimization of large-scale application protocols, and evaluation of the consistency and persistence of *S. phaeochromogenes* ISE-4 under agricultural production systems. Such studies will be essential to determine whether the beneficial effects observed under controlled conditions can be translated into a reliable bioinoculant for cereal and forage crop production.

## Conclusions

To the best of our knowledge, this work provides the first functional characterization of bacterial endophytes isolated from *T. intermedium* based on evaluation of PGP traits and phenotype profiling. By integrating functional characterization of bacterial strains, plant inoculation experiments, and qPCR-based quantification of bacterial abundance, this work reflects a common screening scenario that aims to select candidate bioinoculants based on functional traits and is applied across different plant species. Our work highlights that, in a heterogeneous screening setting involving strains of diverse ecological origins, the host genotype was a major determinant of inoculation outcome, while in vitro PGP traits and colonization abundance were not reliable predictors across the tested host–strain combinations. Future studies should determine whether the beneficial effects of *S. phaeochromogenes* ISE-4 observed under controlled conditions can be reproduced under field conditions and translated into a reliable bioinoculant strategy for sustainable cereal and forage crop production.

## Supplementary Information

Below is the link to the electronic supplementary material.

Supplementary Material 1

Supplementary Material 2

Supplementary Material 3

## Data Availability

The datasets generated and/or analyzed during the current study are available from the corresponding author upon reasonable request.
