# Supplementary material for: Uncoupling traits, colonization, and benefits: genotype-dependent growth promotion by endophytic bacteria in Poaceae, including perennial Kernza
Source: World J Microbiol Biotechnol. 2026 Nov 14;42(12):672. doi: 10.1007/s11274-026-05405-y (PMC13769744; doi:10.1007/s11274-026-05405-y)
Supplement: Supplementary file 1 [file 11274_2026_5405_MOESM3_ESM.docx]

Title: **Uncoupling traits, colonization, and benefits: genotype-dependent growth promotion by endophytic bacteria in Poaceae, including perennial Kernza**

Authors: Jagoda Szydło*, Bliss Ursula Furtado, Edyta Deja-Sikora, Sonia Szymańska, Katarzyna Hrynkiewicz*

*Corresponding Authors: Jagoda Szydło [jagodaszydlo08@gmail.com](mailto:jagodaszydlo08@gmail.com) Katarzyna Hrynkiewicz [hrynk@umk.pl](mailto:hrynk@umk.pl)

**Table S3.** Effect of plant variety, bacterial strain, and variety x strain interaction on plant growth parameters assessed with PERMANOVA (permutational multivariate analysis of variance).

|  | **Df** | **Sum of Squares** | **R^2^** | **F** | **Pr (>F)** |
| --- | --- | --- | --- | --- | --- |
| **Variety** | 4 | 3086.58764 | 0.50237429 | 309.94478 | 0.001 |
| **Strain** | 5 | 175.401349 | 0.0285484 | 14.0905722 | 0.001 |
| **Variety x Strain** | 20 | 404.832093 | 0.06589064 | 8.13037624 | 0.001 |
| Residuals | 995 | 2477.17892 | 0.40318667 |  |  |
| Total | 1024 | 6144 | 1 |  |  |

**Figure S1.** Heatmap represents phenotypic profiles for 94 substrates after incubation for 60 h (*P. lini* FR-P-R2-32, *P. stutzeri* ISE-12, *Pantoea sp.* GT2-2-ED1) or 96 h (*S. phaeochromogenes* ISE-4). The utilization of the substrates is depicted by color intensity (OD_590_), provided on the legend next to the figure.

**Figure S2.** Photos show the growth of five different Poaceae varieties (Kernza, Tetradry, Antonella, Eskapada, Euforia), non-inoculated (control) and inoculated with selected bacterial endophytes.

**Figure S3.** Principal coordinate analysis (PCoA) based on PERMANOVA results showing the effect of plant variety (A) and bacterial strain (B) on plant growth parameters.

**Figure S4.** Absolute bacterial abundance in shoots of five different Poaceae varieties. Different marks denote significant differences (p <0.05, Kruskal-Wallis test) between control (non-inoculated plants) and inoculation with bacterial endophyte.

**Figure S5.** Absolute bacterial abundance in roots of five different Poaceae varieties. Different marks denote significant differences (p <0.05, Kruskal-Wallis test) between control (non-inoculated plants) and inoculation with bacterial endophyte.

**Figure S6.** Linear regression models showing the effects of endophyte abundance and treatment (plant variety, endophyte inoculation) on root fresh weight (FW). Significant differences between plant varieties (Antonella, Eskapada, Euforia, Tetradry) and reference (Kernza) are marked by (*).

**Figure S7.** Linear regression models showing the effects of endophyte abundance and treatment (plant variety, endophyte inoculation) on shoot fresh weight (FW). Significant differences between plant varieties (Antonella, Eskapada, Euforia, Tetradry) and reference (Kernza) are marked by (*).

**Figure S8.** Linear regression models showing the effects of endophyte abundance and treatment (plant variety, endophyte inoculation) on root length. Significant differences between plant varieties (Antonella, Eskapada, Euforia, Tetradry) and reference (Kernza) are marked by (*).

**Figure S9.** Linear regression models showing the effects of endophyte abundance and treatment (plant variety, endophyte inoculation) on shoot length. Significant differences between plant varieties (Antonella, Eskapada, Euforia, Tetradry) and reference (Kernza) are marked by (*).
